# Even minimal student-instructor interactions may increase enjoyment in the classroom: Preliminary evidence that greeting your students may have benefits even if you can’t remember their names

**DOI:** 10.1371/journal.pone.0288166

**Published:** 2023-08-10

**Authors:** Gillian M. Sandstrom

**Affiliations:** 1 School of Psychology, University of Sussex, Brighton, United Kingdom; 2 Department of Psychology, University of Essex, Colchester, United Kingdom; National Sun Yat-sen University, TAIWAN

## Abstract

Students value rapport with their instructors, and benefit from interacting with them; student-instructor contact is related to persistence, satisfaction, grades, etc. Instructors who wish to build rapport with their students are often encouraged to address their students by name. However, learning names is difficult for many people, and when classes are large, or team-teaching restricts the time spent with a group of students, it is even more difficult. Outside the classroom, even minimal social interactions with strangers (e.g., making eye contact, having a brief chat) can increase feelings of connection. Could minimal social interactions between instructors and students also have benefits? A rapport-building intervention was tested on students in three classes taught by two instructors (*N* = 352). Compared to students in a control condition and students who were assigned to a greeting condition (a minimal interaction designed to enable instructors to recognize students’ faces) reported a stronger relationship with the instructor, and greater relationship strength predicted greater interest/enjoyment, relatedness and belonging. This novel intervention produced similar results to a more traditional nameboard condition, designed to enable instructors to learn students’ names. These findings raise the intriguing possibility that even when instructors struggle to learn students’ names, they can still build rapport with their students by simply greeting them as they enter class.

## Introduction

Students are more likely to learn and develop intellectually when they have effective instructors. But what defines an effective instructor? Researchers have proposed two key factors: 1) the instructor’s skill and 2) their rapport with students, factors that have also emerged in student evaluations [1, 2]. This is unsurprising, given that competence and warmth have been proposed as universal dimensions of the judgments we make about others [3]. According to one study, instructors who demonstrate both skill and rapport are 96% likely to be judged positively overall, whereas those who demonstrate only skill or only rapport are less likely to be judged positively overall (59% and 67% respectively; [1]). Students and instructors do not always agree on the relative importance of skill and rapport for effective teaching [2]. One review found that across 30 studies, whereas instructors placed more importance on skill (e.g., being intellectually challenging, setting high standards), students placed more importance on rapport (e.g., helpfulness and availability) [4].

The concept of *rapport* is pervasive in the pedagogical literature, but the way that rapport has been operationalized has varied. It is thought to include behaviors such as showing empathy and concern, welcoming students to ask questions and contribute to discussions, and expressing an interest in students as individuals [2, 5–7]. One conceptualization of rapport, not specifically related to instructor-student relationships, identifies three components: mutual attentiveness, positivity, and coordination [8]. In terms of rapport between instructors and students, one of the key features seems to be a sense of connection, and therefore the current research measures students’ perceptions of the strength of their relationship with the instructor.

Students who perceive a strong relationship with their instructors experience a wide range of benefits. Student-instructor interactions have been related to: more positive attitudes towards the instructor and the class [7, 9], satisfaction with education [10, 11] (but see [12]), belonging [13], attendance [7], paying attention and studying more [7], degree aspiration [10, 14], retention [15, 16], engagement [17], and grades [10, 14, 18]. These effects tend to hold after controlling for student background and institutional characteristics, though they sometimes vary in strength depending on gender, race and social class. A separate, but related, line of research finds that verbal and non-verbal “immediacy” behaviors, which build and express the instructor-student relationship, also have benefits: more positive attitudes towards the instructor and the class, motivation, enjoyment, and perceived learning [19, 20].

Instructors build and convey their relationship with their students through the use of non-verbal “immediacy” behaviors such as smiling, gesturing, and moving around the classroom while they are teaching, and through verbal behaviors such as talking about personal experiences, using humor, and soliciting questions from students [21]. Instructors are often encouraged to adopt two particular verbal immediacy behaviors. First, a top tip for building rapport is to learn students’ names [21–24]. Instructors have developed several methods to facilitate this (e.g., studying flashcards with students’ pictures/names, creating a seating chart and asking students to always sit in the same place) [23, 25]. Second, instructors are often encouraged to talk to their students. Many higher education institutions have invested time and money to provide opportunities for instructors and students to interact [26]. These student-instructor interactions can take many forms. One observational study at a residential college in the United States documented four types of interactions: incidental contact (e.g., greetings, waves), functional interactions (e.g., talking about class material), mentoring, and personal interactions [27]. Other studies have distinguished between the formal interactions that contribute to academic integration (e.g., talking about course content or career plans) and the informal interactions that contribute to social integration (e.g., talking about personal problems, socializing).

Although the pedagogical literature is replete with studies on how to strengthen instructor-student relationships, in the actual context of academic life, the behaviors that lead to connection can be quite rare. One intuitive factor is the reluctance of overworked instructors to spend the time and effort required, but research finds more evidence for the role of instructors’ values and beliefs about the importance of teaching undergraduates [28]. Instructors’ attitudes include “my students don’t appreciate how much I care” (p. 206), “I don’t want to get too close/I can’t get close” (p. 207), and “my job is to teach, not to care” (p. 207) [2]. In other words, instructors may not see interacting and connecting with students as an important part of their job, possibly because they are not aware that students’ feelings of connection contribute to their learning.

Even when instructors do acknowledge that building a connection helps students learn, there are many reasons why they may not engage in relationship-building behaviors. Although a top tip for building a connection is to learn students’ names, many instructors profess difficulties with faces and names. Indeed, learning names is a difficult cognitive task, because names lack the semantic networks that people have for other words [29]. It may be especially difficult when team-teaching limits how often instructors see the same students, and when class sizes are large (although, see [23]). Indeed, student evaluations of rapport are negatively related to class size [5, 30]. Finally, instructors may worry about misremembering or mispronouncing names, especially names that they are not familiar with (e.g., students from diverse backgrounds).

Not only do instructors find it difficult to learn names, but they may also struggle with another highly promoted verbal immediacy behavior: talking to students. Instructors (and students) may feel apprehensive about how they will be judged, and feel unsure about what to say [31]. Indeed, these are common fears in other social situations, such as talking to a stranger [32]. Social interactions are challenging for many people, and the difficulties are exacerbated when there are age or other status differences between the parties who are interacting. One study finds that instructors’ discomfort with the social dynamics of student relationships is the single biggest factor inhibiting instructors from engaging with students [28]. Further, instructors and students may not even recognize opportunities for interaction; research has found that student-instructor interactions are rare [33], even when circumstances result in instructors and students being in the same room [27].

Given the challenges instructors face in following two common pieces of advice for building relationships with students (i.e., learning names, talking to students), the current research tests an alternative strategy: greeting students as they arrive to class. Recent research in social psychology has found that even minimal social interactions can help people feel more connected [34]. Researchers have found that people experience a more positive mood and feel more connected when they talk to acquaintances [35], the barista at the coffee shop [36], or even a stranger, during the morning commute [37, 38]. Feelings of connection can occur even in the absence of talking; people feel less disconnected when they merely make eye contact with the people they walk past [39]. Might students also benefit from minimal social interactions? Could simply greeting students at the door convey the attentiveness and positivity that contribute to feelings of rapport [8]?

In the current study, I test a novel relationship-building intervention: greeting students as they arrive to class (to allow the instructor to recognize students). I compare this to a more traditional intervention: having students display nameboards (to allow the instructor to learn students’ names). I predicted that both of these interventions would increase students’ feelings of interest/enjoyment, relatedness and belonging. The study was pre-registered on the Open Science Framework (OSF; https://osf.io/85hg2) after data from one of three samples were analyzed. The materials, data and analysis script are available on OSF (https://osf.io/r2vgw/). Please see the Supplemental Materials (also on OSF at the same link) for a Transparency Statement detailing deviations from the pre-registration.

## Method

This study was granted ethical approval by the Faculty of Science and Health Ethics Committee at the University of Essex. All participants provided informed consent in writing.

### Procedure

Students were recruited from my second-year undergraduate statistics lab classes in two separate academic years (samples 1 and 2), and from a colleague’s first-year undergraduate statistics lab class (sample 3; see Table 1). Due to team teaching, I taught only for the first five weeks of the year, so the intervention was delivered over these five weeks, and all students who attended the lab class during the 5^th^ week of the term were invited to participate in the study.

**Table 1:** Sample details.

Sample	Student Year	Acad. Year	Instr.	Greeting N	Nameboard N	Control N	Total	Class Size	% Class
**1**	2	2016	G.S.	38	32	37	107	198	54%
**2**	2	2017	G.S.	50	56	22	128	215	60%
**3**	1	2017	X	32	27	58	117	238	44%
**Total**				120	115	117	352	651	54%

The instructor was the first author (G.S.) for samples 1 and 2, and someone else (X) for sample 3.

All students in each sample attended the same lecture each week, but were split by the university’s timetabling system into smaller groups for lab classes. Each lab class was quasi-randomly assigned to condition (see Supplemental Materials on OSF for details on condition assignment). The manipulation and measurements were all carried out in the lab classes, and not the lectures. During all lab classes, students worked independently on computers, and raised their hand if they had questions. The instructor, and a team of graduate teaching assistants, circulated to answer questions. In samples 1 and 2, the lectures were taught by the same instructor as the lab classes, but in sample 3, the lectures were taught by a different instructor than the lab classes.

The lab classes for each sample were exposed to one of three conditions: greeting, nameboard, or control. In the greeting condition, the instructor stood at the door, and greeted students as they entered the computer lab (e.g., saying “good morning”). In the nameboard condition, students were provided with a nameboard (also known as a name card, or name tent) to place on the desk in front of them, on which they wrote their name. In the control condition, students did not have nameboards and were not greeted at the door as they entered class. Note that this is not a reduced contact condition, but rather a condition that reflects the usual situation for students: students in this department at this university do not normally have nameboards, and the instructors do not normally stand at the door and greet students.

Although students were assigned to a particular lab class, and should therefore be exposed to the same condition for five weeks, they sometimes switched lab classes, either officially or unofficially. Therefore, students were asked to report which lab class they attended during each of the first 5 weeks. They were included in the analysis if they reported attending the same lab class (i.e., were subject to the same manipulation) at least three times. For example, if a student attended a lab class with nameboards twice, and attended a lab with greetings three times, they would be considered in the greeting condition. A total of 27 students who were retained in the analysis reported having been exposed to more than one condition. Results are unchanged if these students are removed from the analyses (see the Supplemental Materials on OSF). A further 35 students were not exposed to any condition at least three times (e.g., due to switching lab classes, or not attending), and were excluded from analyses.

### Participants

A total of 387 students completed the survey in week 5. The remaining students in each class did not participate in the study. A small number of students (exact number unknown) attended the lab class in week 5, but chose not to participate in the study. Non-participation was mainly due to students not attending the lab class in week 5. I intended to track attendance for the classes that I taught (i.e., samples 1 and 2), but neglected to log attendance in week 5. However, as an indicator of how many students attend (and do not attend) lab classes each week, it may be helpful to know that during the previous week, 65% of sample 2 attended class, suggesting that the 60% of sample 2 who participated in the study in week 5 constituted the vast majority of students who attended that week.

A total of 35 students were exposed to a condition fewer than three times and were not included in the analyses, leaving a final sample of 352. A total of 27 students (8% of the final sample) were exposed to multiple conditions, but were included in the analyses as per pre-registered criteria. See Procedure for details about these two groups of participants.

In the final sample, 280 students (79.5%) identified as female, 69 (19.6%) as male, and 3 (0.9%) as non-binary. A sensitivity analysis using G*Power [40] suggests that our final sample (*N* = 352) can detect effects where *f* ≥ 0.17 (i.e., a small-medium effect).

### Measures

#### Instructor-student relationship

Students were asked 1) whether they had ever spoken to the instructor, 2) whether they thought the instructor recognized them, and 3) whether they thought the instructor knew their name. For example, students responded Yes or No to “[The instructor] knows my name” (sample 1) or “Do you think [the instructor] knows your name?” (samples 2 and 3).

I created a new variable called *relationship strength*, based on responses to this set of questions. If a student thinks the instructor knows their name, I set relationship strength to 2. If a student thinks the instructor recognizes them, but does not know their name, then I set relationship strength to 1. If neither of these is true (i.e., they think the instructor does not recognize them), I set relationship strength to 0.

#### Interest/Enjoyment

Students’ interest and enjoyment in class were measured with seven items (two reverse-scored), measured on a scale from 1 = *not at all true* to 7 = *very true*. These were based on the items in the interest/enjoyment subscale of the Intrinsic Motivation Inventory (IMI) [41], but modified to be relevant to a class (e.g., “This class is fun to be a part of”, “I think this class is boring”). These items showed good reliability (α = .89), and were averaged.

#### Relatedness and belonging

Eight items (four reverse-scored) were adapted from the relatedness subscale of the IMI (e.g., “I’d like a chance to interact with people in this class more often”, “I feel really distant from people in this class”). These items showed good reliability (α = .73), and thus were averaged into a relatedness composite.

In addition, two face-valid questions (measured on the same scale) were included: “I feel like I belong in this class”, and “I feel like I belong at [this university]”. These two items were significantly correlated, *r*(351) = .32, *p* < .001, and thus were averaged into a belonging composite.

The relatedness and belonging composites were correlated with each other, *r*(352) = .46, *p* < .001, but not at the cut-off specified in the pre-registration, thus were analyzed separately.

## Results

The analyses that follow were pre-registered, but there are some minor deviations from the pre-registration, which are discussed in the Supplemental Materials (see OSF).

### Descriptives

The dependent variables were moderately correlated with each other; interest/enjoyment was moderately correlated with both relatedness, *r*(352) = .32, *p* < .001, and belonging, *r*(352) = .41, *p* < .001, which were moderately correlated with each other, *r*(352) = .46, *p* < .001. The moderate sizes of these correlations suggest that the outcome measures are independent, rather than being different aspects of the same construct.

### Manipulation check: Do the two interventions strengthen instructor-student relationships?

The two interventions were designed to strengthen instructor-student relationships: the novel greeting condition was intended to help instructors recognize students, and the traditional nameboard condition was intended to help the instructor learn students’ names.

Consistent with my pre-registered hypotheses, Chi-square analyses found that more students in the greeting condition (48%) thought that the instructor recognized them, compared to the control condition (25%), χ^2^(1) = 13.80, *p* < .001. Further, more students in the nameboard condition (31%) thought that the instructor knew their name, compared to the control condition (7%), χ^2^(1) = 23.06, *p* < .001. In other words, the interventions seem to have worked as expected.

### Do the two interventions directly increase interest/enjoyment, relatedness and belonging?

**Fig 1:**
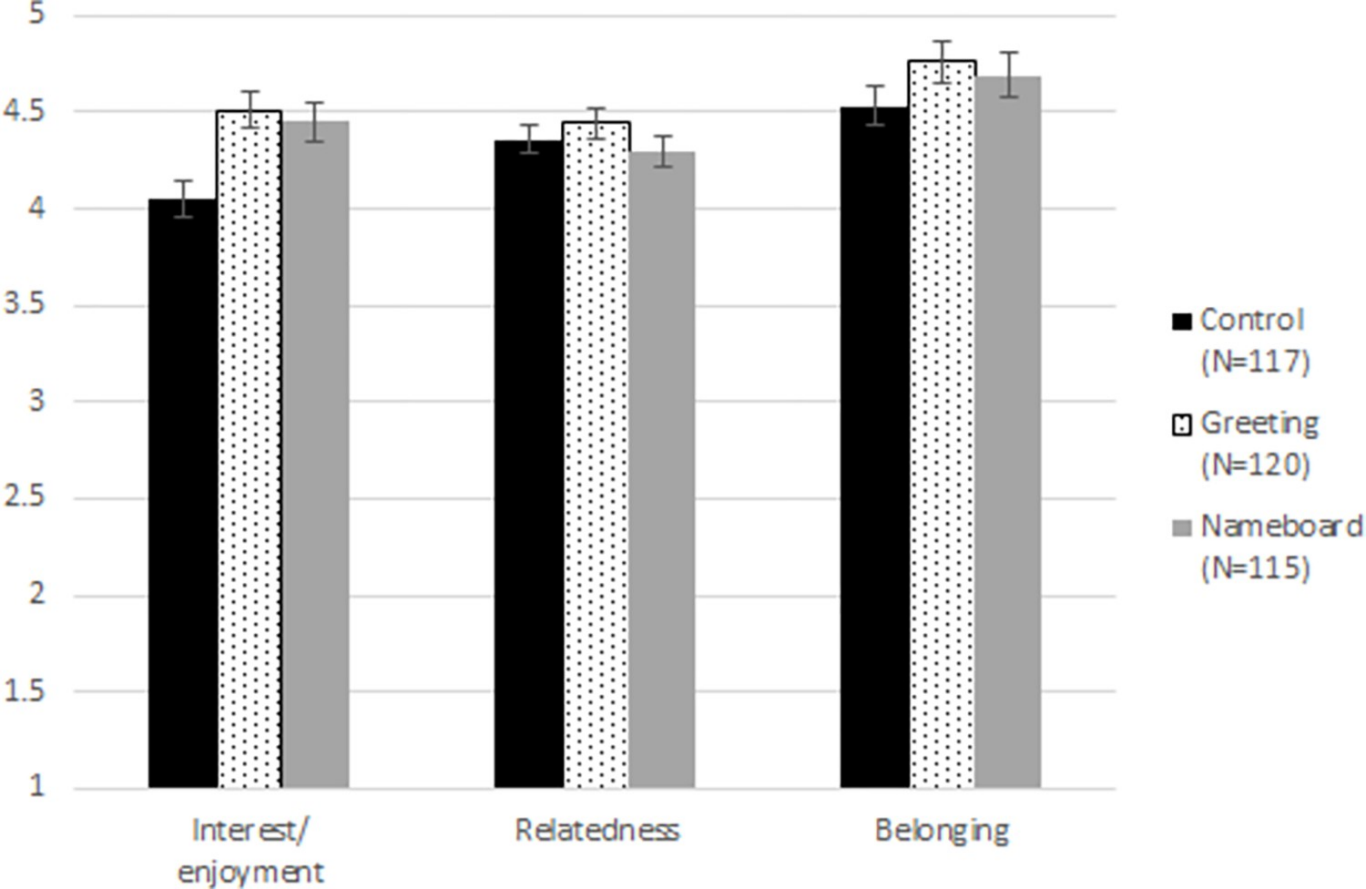
Students’ interest/enjoyment and belonging, by condition. Bars indicate standard error of the means.

Given that both interventions (greeting students as they arrive to class, and having students display nameboards) increased relationship strength, they would also be expected to increase interest/enjoyment, relatedness and belonging, which have been associated with rapport in past studies. To test this, I ran a one-way ANOVA, including sample as a fixed effect, to control for differences between samples. I found a significant effect of condition on interest/enjoyment, *F*(2, 347) = 4.39, *p* = .01, η^2^ = .03 (see Fig 1). Post-hoc Tukey’s tests revealed that students reported more interest/enjoyment in both the greeting (*M* = 4.51, *SD* = .98) and nameboard conditions (*M* = 4.45, *SD* = 1.05), compared to the control condition (*M* = 4.05, *SD* = 1.05), *p*’s < = .01. The two intervention conditions did not differ from each other, *p* = .88. Contradicting the hypotheses, there was no direct effect of condition on relatedness, *F*(2, 347) = 0.88, *p* = .42, η^2^ = .01, or belonging, *F*(2, 347) = 0.88, *p* = .41, η^2^ = .01.

In keeping with the spirit of my pre-registration, I also tested separate models for each student year, which is confounded with instructor, since one instructor–the author–taught second-year students and the other instructor taught first-year students. The direct effect of condition on interest/enjoyment was directionally significant for the second-year students, who were taught by the author, *F*(2, 232) = 2.76, *p* = .07, η^2^ = .02, but not significant for the first-year students, who were taught by a different instructor, *F*(2, 114) = 1.42, *p* = .25, η^2^ = .02.

### Do the two interventions indirectly increase interest/enjoyment, relatedness and belonging, by increasing relationship strength?

Although condition did not have a direct effect on relatedness and belonging, it is possible that the interventions worked *to the extent that* they resulted in the instructor recognizing students or knowing their names. Recall from the manipulation check analyses that condition predicted relationship strength as expected. Next, I tested whether relationship strength has a direct effect on the outcome measures. This analysis was pre-registered as an alternative to the main analysis.

**Fig 2:**
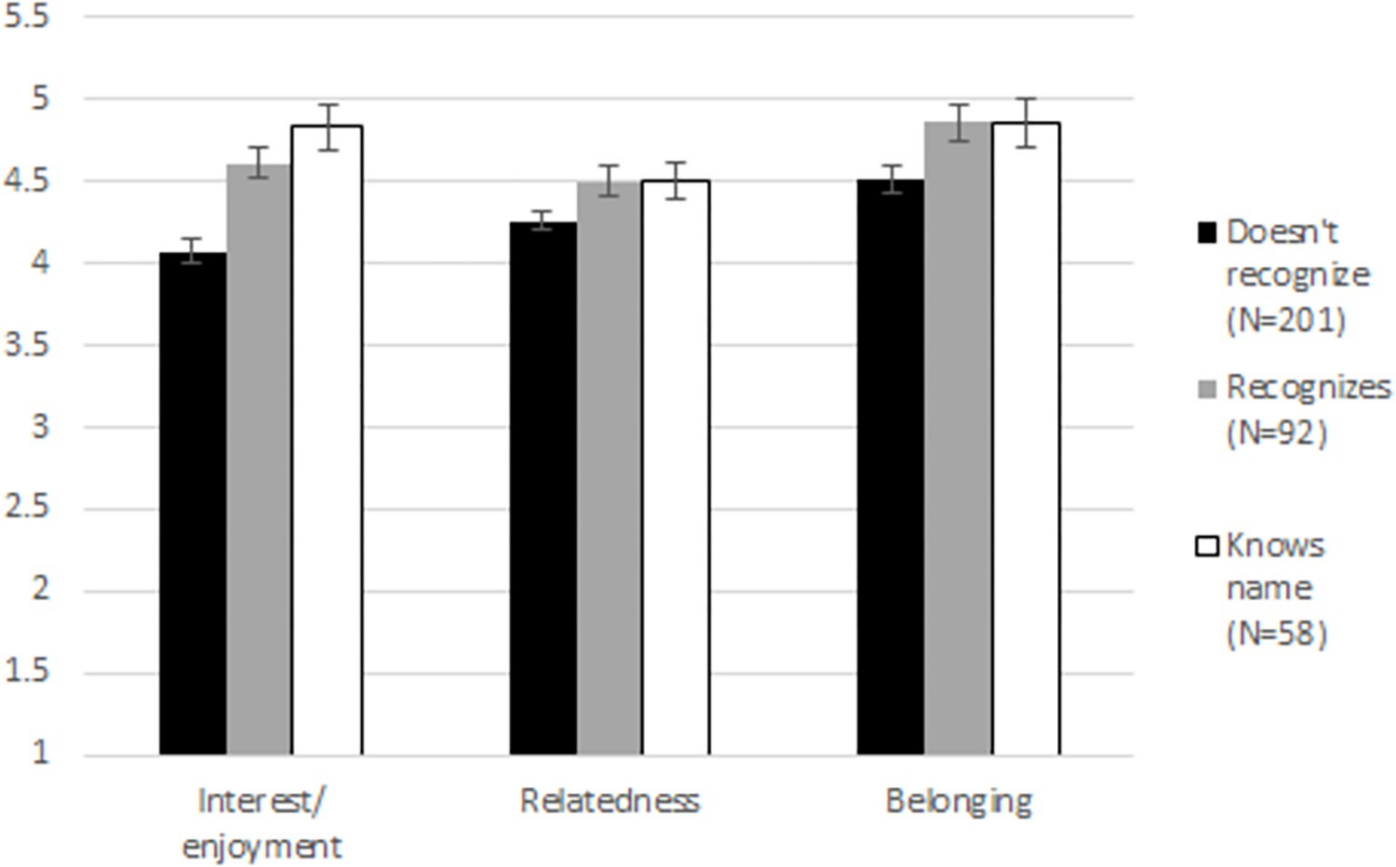
Students’ interest/enjoyment, relatedness and belonging, by relationship strength. Bars indicate standard error of the means.

To test this, I again ran a one-way ANOVA, including sample as a fixed effect, to control for differences between samples. I found a significant effect of relationship strength on interest/enjoyment, *F*(3, 346) = 11.06, *p* < .001, η^2^ = .06, consistent with the direct effect of condition on this measure (see Fig 2). Post-hoc Tukey’s tests revealed that students reported more interest/enjoyment when they thought the instructor knew their name (*M* = 4.83, *SD* = 1.03), or thought the instructor recognized them (*M* = 4.61, *SD* = .88), compared to when they thought the instructor did not recognize them (*M* = 4.07, *SD* = 1.04), *p*s < .001. There was no difference in interest/enjoyment between students who thought the instructor knew their name and students who thought the instructor recognized them, *p* = .39.

Although there were no direct effects of condition on relatedness or belonging, there were significant direct effects of relationship strength on both relatedness, *F*(3, 346) = 6.75, *p* = .001, η^2^ = .04, and belonging, *F*(3, 346) = 3.35, *p* = .04, η^2^ = .02. For relatedness, post-hoc Tukey’s analyses revealed no significant differences between conditions, though students who thought the instructor did not recognize them had the lowest mean. Post-hoc Tukey’s analyses revealed that students who thought that the instructor did not recognize them reported lower belonging (*M* = 4.51, *SD* = 1.17) than students who thought the instructor recognized them (*M* = 4.86, *SD* = 1.05), *p* = .04, but not significantly lower belonging than students who thought the instructor knew their name (*M* = 4.86, *SD* = 1.09), *p* = .09.

I again looked at student year (confounded with instructor) as a possible moderator. The main effects of relationship strength on interest/enjoyment, relatedness and belonging were significant for the second-year students, who were taught by the author (marginal for belonging, *p* = .06), but not for the first-year students, who were taught by a different instructor, *p*’s > .25.

In sum, condition predicts relationship strength, and relationship strength predicts interest/enjoyment, relatedness and belonging. This is consistent with the idea that there may be an indirect effect of condition on the DV’s, via relationship strength. I tested this by using model 4 of PROCESS (with 10,000 resamples) [42], with condition (nameboard or greeting = 1; control = 0) entered as X, interest/enjoyment, relatedness and belonging (entered separately) as Y, and relationship strength (instructor knows name = 2; instructor recognizes = 1; instructor does not recognize = 0) as M. There were significant indirect effects of condition, via relationship strength, on interest/enjoyment, β = .20, *SE* = .07, CI_95_ = [.09, .35], relatedness, β = .12, *SE* = .05, CI_95_ = [.04, .22], and belonging, β = .10, *SE* = .05, CI_95_ = [.01, .22]. Given that relationship strength is categorical, these indirect effects should be interpreted cautiously.

## Discussion

The current research complements a wealth of studies documenting how students benefit from interactions with instructors. The results of this preliminary study show that not only do students benefit when instructors know their name, but they may also benefit when instructors simply greet them as they arrive to class. These results suggest a possible means to counteract the drop in rapport that has previously been observed as class sizes increase [5, 30], and will be welcome news to instructors who struggle to learn student names, including those teaching large classes. However, there are some important limitations (see discussion below), and further work is needed to validate the results and understand exactly what conditions allow these effects to occur.

The current study has implications for the growing literature on minimal social interactions. Past research has found that talking to strangers, acquaintances and service providers, or even just making eye contact with a stranger, results in a better mood and greater feelings of social connection [32, 35–39]. Developing close relationships with others can be time-consuming and difficult, so it is valuable to know that even minimal social interactions can go some distance to fulfilling the human need to belong [34]. The current study examines a new context, finding that minimal social interactions in the classroom, between instructors and students, result in similar outcomes: instructors simply greeting students at the door resulted in more interest/enjoyment in the class, and greater feelings of relatedness and belonging. Developing relationships with students can be time-consuming and difficult, especially when team-teaching or teaching a large class, so it is valuable to know that in a classroom context too, minimal social interactions have benefits.

Greeting students at the door has several advantages compared to learning students’ names. As reviewed in the introduction, learning students’ names can be difficult for a large number of reasons, including cognitive constraints, motivation, and large class sizes. Further, instructors may worry that they will have difficulty learning some names (e.g., from a different cultural context), mispronounce a name, or address a student by the wrong name, and that this could potentially damage feelings of rapport. (I am not aware of any research suggesting whether or not this fear is well-founded.) I am not suggesting that instructors should not attempt to learn students’ names, and several methods have been developed to help instructors do so (e.g., face-name flashcards, seating charts; see [23, 25]). However, if instructors do not have the time or motivation, the current results suggest an alternative for developing rapport with students, and providing a benefit to their students.

Can any instructor stand at the door, greet their students, and see these positive effects? The literature on immediacy suggests that instructors convey their psychological availability to students through a variety of verbal and nonverbal behaviors. Acknowledging students by making eye contact and greeting them might be considered a kind of immediacy behavior. If an instructor genuinely wants to connect with their students, then greeting students at the door will be consistent with other signals that they send via their immediacy behaviors, and there is no reason to doubt that students will appreciate the effort and reap the benefits. However, if an instructor stands at the door and unconvincingly delivers a perfunctory greeting, without exhibiting other immediacy behaviors, then students would have reason to doubt the authenticity of the attempt at relationship-building, and are unlikely to experience any benefits.

Will greetings be effective in any class? Research is needed to determine the generalizability of the findings (see limitations, below). Given that it is especially difficult to build rapport in large classes [5, 30], it seems encouraging that greeting students had positive benefits in large classes in this study (approximately 200 students in each sample, though divided into smaller groups for the lab classes). It is possible though, that when the class size is smaller, students would *expect* instructors to recognize them and know their names, and therefore a simple greeting might not have as large an effect. However, even with small classes, and even if instructors know all of their students’ names, relationships must be actively maintained or they dissipate. Greeting students at the beginning of each class seems like one way for instructors to repeatedly convey their psychological availability to students.

The results of the current study suggest that learning students’ names and greeting students at the door are two effective ways to increase relationship strength, but obviously there are many other ways (both student-initiated and instructor-initiated) to increase relationship strength as well. For instance, in this study, the author recognized some of the students in the control condition (e.g., students who frequently asked questions), and knew the names of some of the students in the control and greeting conditions (e.g., some were students that had volunteered in her lab). These pre-existing relationships weaken the purity of the relationship between the condition assignment and relationship strength in this study; the fact that the results came through despite this speaks to the strength of the effect.

On a related note, it is worth pointing out that the questions about relationship strength were students’ perceptions, which might not match reality; some students might think that the instructor recognizes them or knows their name even when the instructor does not, or think that the instructor does not recognize them or know their name when the instructor actually does. Indeed, while completing the survey, multiple students asked the author if she knew their names (and were delighted to learn that she did!) In one recent study, instructors knew fewer names than students thought they did [43]. That study concluded that students’ perceptions of being known by name may be more important than the reality. Similarly, anecdotal evidence suggests that feeling like instructors are making an effort to learn names is more important to students than whether or not the instructor knows their name [23], an opinion which is shared by the author.

### Limitations and future research

More research is needed to establish the generalizability of the findings. The current study examined data gathered in only two classes at one university. Of further concern, there were some differences between the results in those two classes. It is possible that these differences suggest limitations to generalizability, but there are several other reasons that might explain these differences.

First, there were differences in how those classes were taught. For the second-year samples, the instructor (i.e., the author) taught both the weekly lecture and attended the weekly computer lab class, whereas for the first-year sample, one person taught the lecture and a different person attended the lab. It is possible that the second-year students felt safer talking to and/or were more motivated to develop a relationship with the author, precisely because they saw her more often. Indeed, there was a large difference between the number of students who had ever spoken to the instructor (i.e., the author) in the second-year samples (82%) compared to the number of students who had ever spoken to the instructor in the first-year sample (42%), let alone in the number who felt like they were recognized (58% vs. 12%) or known (24% vs. 3%). In particular, the number of students in the first-year sample who felt like the instructor knew their name was so small (*n* = 3) that it is statistically questionable to even test the effect.

A second reason that might explain the different results between classes is that one of those classes was first-year students and one was second-year students. It may be that first-year students in their first few weeks of class need some time to work up the courage to ask questions, or recognize this as the norm in these lab classes. First-year students might be so preoccupied with the adjustment to university life that they don’t notice attempts at rapport-building, or don’t feel comfortable with the idea of establishing rapport with the instructor.

Finally, the two classes that were studied might have shown different results because they were taught by different instructors (i.e., the student year was confounded with the instructor), and the instructors had different ways of interacting with their students. In the nameboard condition, when a student asked the author a question, she attempted to learn the student’s name. If there was a slow period when no students were asking questions, the author would occasionally walk around the room studying students’ names. The other instructor might not have done either of these things–they didn’t receive any instructions to do so.

Further, the instructors were not blind to condition or hypotheses. Ideally, we would study the effects of greeting students in many classes, with many instructors, who are all blind to the hypothesis. In practice, however, having instructors be blind to the hypothesis would be difficult; I think most instructors, if asked to greet students at the door, would assume that the experimenter expected to see positive outcomes. Recruiting a large number of instructors would obviously be logistically difficult. And it would have to be a large number, otherwise one might ask whether there was something special about a particular instructor or a particular class that might affect the outcomes. Every study design has strengths and weaknesses. I chose to capitalize on the fact that the class I was teaching assigned students to one of three different lab classes, which allowed me to reduce variance by keeping the class content and the instructor constant across all three conditions.

Although the current study design held the instructor (e.g., personality, teaching style) constant across conditions, it is possible that, because the instructors were not blind to condition or hypothesis, the same instructor behaved differently in the different conditions. For example, maybe when instructors greeted students at the door, students greeted them back, and smiled, and this put the instructors in a better mood, which resulted in them being more helpful during the lab. To some extent, it doesn’t matter if this is the case, as long as all instructors are likely to have their behaviour affected in a similar way. But clearly more research is needed to determine whether the key factor underlying the effectiveness of the greeting manipulation is the greeting itself, how the instructor’s behaviour changes because of the greeting, or a combination of these, and potentially other, factors.

## Conclusion

The most effective instructors build a personal connection with their students, which produces wide-ranging benefits (e.g., belonging, attendance, engagement, and grades; see Introduction). Students benefit when instructors know their names, but when that is difficult, simply greeting students as they arrive to class may help instructors build a relationship with their students, which allows the students to enjoy similar benefits: interest/enjoyment in class, and feelings of belonging.

## Supporting information

S1 File Supplemental materials. Transparency statement, additional details on methods, and analyses with participants who experienced conflicting conditions excluded can be found on the Open Science Framework here: https://osf.io/r2vgw/.
(DOCX)
