# Supplementary material for: Even minimal student-instructor interactions may increase enjoyment in the classroom: Preliminary evidence that greeting your students may have benefits even if you can’t remember their names
Source: PLoS One. 2023 Aug 10;18(8):e0288166. doi: 10.1371/journal.pone.0288166 (PMC10414558; doi:10.1371/journal.pone.0288166)
Supplement: S1 File — Transparency statement, additional details on methods, and analyses with participants who experienced conflicting conditions excluded can be found on the Open Science Framework here: https://osf.io/r2vgw/. (DOCX) [file pone.0288166.s001.docx]

**S1 File. Supplemental materials.** Transparency statement, additional details on methods, and analyses with participants who experienced conflicting conditions excluded can be found on the Open Science Framework here: https://osf.io/r2vgw/.
